# Effect of Humin and Chemical Factors on CO_2_-Fixing Acetogenesis and Methanogenesis

**DOI:** 10.3390/ijerph19052546

**Published:** 2022-02-22

**Authors:** Biec Nhu Ha, Duyen Minh Pham, Takuya Kasai, Takanori Awata, Arata Katayama

**Affiliations:** 1 Department of Civil Engineering, Graduate School of Engineering, Nagoya University, Chikusa, Nagoya 464-8603, Japan; ha.nhu.biec.e5@s.mail.nagoya-u.ac.jp (B.N.H.); kasai.takuya@imass.nagoya-u.ac.jp (T.K.); 2 Institute of Materials and Systems for Sustainability, Nagoya University, Chikusa, Nagoya 464-8603, Japan; pham.duyen.minh@imass.nagoya-u.ac.jp; 3 Graduate School of Engineering, Osaka Institute of Technology, Osaka 535-8585, Japan; takanori.awata@oit.ac.jp

**Keywords:** humin, CO_2_-fixing acetogenesis, CO_2_-fixing methanogenesis, iron sulfide, sulfide, sodium ion

## Abstract

Acetogenesis and methanogenesis have attracted attention as CO_2_-fixing reactions. Humin, a humic substance insoluble at any pH, has been found to assist CO_2_-fixing acetogenesis as the sole electron donor. Here, using two CO_2_-fixing consortia with acetogenic and methanogenic activities, the effect of various parameters on these activities was examined. One consortium utilized humin and hydrogen (H_2_) as electron donors for acetogenesis, either separately or simultaneously, but with a preference for the electron use from humin. The acetogenic activity was accelerated 14 times by FeS at 0.2 g/L as the optimal concentration, while being inhibited by MgSO_4_ at concentration above 0.02 g/L and by NaCl at concentrations higher than 6 g/L. Another consortium did not utilize humin but H_2_ as electron donor, suggesting that humin was not a universal electron donor for acetogenesis. For methanogenesis, both consortia did not utilize extracellular electrons from humin unless H_2_ was present. The methanogenesis was promoted by FeS at 0.2 g/L or higher concentrations, especially without humin, and with NaCl at 2 g/L or higher concentrations regardless of the presence of humin, while no significant effect was observed with MgSO_4_. Comparative sequence analysis of partial 16S rRNA genes suggested that minor groups were the humin-utilizing acetogens in the consortium dominated by *Clostridia*, while *Methanobacterium* was the methanogen utilizing humin with H_2_.

## 1. Introduction

CO_2_ emissions from the combustion of fossil fuels for energy production and industrial processes are believed to be key contributors to global warming [1,2,3]. While various technologies have been being studied intensively to capture or utilize CO_2_ to reduce the emissions [4], it is also important to understand the carbon cycle on earth, where biological CO_2_ fixation by autotrophic microorganisms plays an important role. In nature, autotrophic microorganisms utilize light or inorganic compounds as an energy source to reduce CO_2_ into organic compounds via six natural carbon fixation pathways including the Calvin–Benson–Bassham cycle, the reductive citric acid cycle, the 3-hydroxypropionate bicycle, the 3-hydroxypropionate/4-hydroxybutyrate cycle, dicarboxylate/4-hydroxybutyrate cycle, and the reductive acetyl-CoA pathway (Wood–Ljungdahl pathway) [5,6,7]. In these CO_2_-fixation pathways, the Wood–Ljungdahl pathway has high energy efficiency due to the consumption of only one mole of ATP to fix one mole of CO_2_ [7], which has garnered more interest for many researchers. In Bacteria, acetogens (e.g., *Clostridium* and *Acetobacterium*) synthesize acetate from CO_2_ and H_2_ via the Wood–Ljungdahl pathway (Equation (1)) [8,9], while in Archaea, methanogens (e.g., *Methanobacterium*) generate methane from CO_2_ and H_2_ via hydrogenotrophic methanogenesis coupled to the Wood–Ljungdahl pathway (Equation (2)) [9,10].

4H_2_ + 2CO_2_ → CH_3_COOH + 2H_2_O (∆Gr = −95 kJ/mole) (1)

4H_2_ + CO_2_ → CH_4_ + 2H_2_O (∆Gr = −131 kJ/mole) (2)

H_2_ is a widely utilized electron donor for fixing CO_2_ by acetogens and methanogens. In the environment, however, H_2_ is not present ubiquitously at high concentration; acetogens and methanogens, therefore, live with syntrophs such as H_2_ producer or utilize other molecules such as humic substances to obtain energy for acetogenesis and methanogenesis [11]. Humic substances (HSs) are naturally occurring organic matter distributed widely in soils and sediments, and their functions as extracellular electron mediators have been reported, especially for microbial iron reduction [12,13]. In a previous report [14], we found that humin, an insoluble fraction of HSs, has an ability to act as an electron donor for CO_2_-fixing acetogenesis and methanogenesis. Humin accounts for about 50–70% of the total HSs on earth [15], and humin extracted from many natural sources has shown stable electron mediating activity [16,17]. Therefore, humin can be a potential alternative electron donor or supporter on biological CO_2_-fixation processes in environments where H_2_ is limited.

There are also chemical factors affecting acetogens and methanogens. Iron sulfide (FeS) is a common soil mineral formed as a precipitate from the reaction between aqueous S (II) and Fe (II) [18]. Both of these are key components in amino acids, vitamins, co-enzymes, co-factors, which are essential to microbial metabolic reactions. Especially, corrinoid Fe-S protein serves as a methyl carrier in the Wood–Ljungdahl pathway [19]. The presence of sulfate influences a syntrophic interaction of acetogens, methanogens, and sulfate-reducing bacteria [20]. Under standard conditions, sulfate reduction is thermodynamically more favorable than methanogenesis and acetogenesis [21,22]. Furthermore, sulfide produced from sulfate reduction is toxic to acetogens and methanogens [23,24]. Salinity (sodium chloride (NaCl)) also affects the microbial CO_2_-fixing activities. Some acetogens and methanogens utilize Na^+^ motive force to make ATP [8,25,26], while NaCl at high concentrations inhibits microbial growth in general.

In paddy soils, humic substances are generally present. FeS, sulfate, and NaCl are also present in paddy soils, especially in or near brackish water areas where there are transition zones from terrestrial to marine environments [27]. Acetogens and methanogens would have multiple and complex interactions with these chemical factors and humin. However, to date, knowledge on the effects of these factors on CO_2_-fixing acetogenic and methanogenic activities in the presence of humin is lacking. Therefore, this study was conducted to evaluate the effects of FeS, magnesium sulfate (MgSO_4_), and NaCl on the acetogenesis and methanogenesis of CO_2_ fixation in mixed cultures in the presence and absence of humin.

## 2. Materials and Methods

### 2.1. Humin Extraction and Preparation of Chemically Reduced Humin

Soil was collected from a paddy field in Kamajima, Aichi Prefecture, Japan, and utilized for humin extraction. Humin was prepared as described previously [28]. Humin contained 1.3% carbon, 0.4% hydrogen, 0.2% nitrogen, 4.3% of oxygen, and 93.9% of ash (Table S1). Sulfur was not detected. Fourier transform infrared (FTIR) spectrum of humin, measured by a JASCO FT-IR-6100 spectrometer (JASCO, Japan), showed the presence of hydroxyl, methylene, aromatic carbonyl, and nitrile groups (Figure S1). Humin characteristics used here were comparable with previous studies [15].

Chemically reduced humin was prepared by adding 5 g of humin to a 250 mL bottle containing 100 mL of anaerobic 0.1 M NaBH_4_ solution with *N*,*N*-dimethylacetamide as the solvent. The mixture was continuously stirred using a magnetic stirrer at room temperature for one week, following which the remaining NaBH_4_ was gently removed by adding acetic acid (1 M) to neutralize the pH of the mixture. The reduced humin was collected on a Whatman glass microfiber filter (47 mm diameter), in vacuo. Humin reduction was carried out entirely in an anaerobic chamber (Coy Vinyl Anaerobic Chamber 7450000; the Coy Laboratory Products, Grass Lake, MI, USA).

### 2.2. Microbial Consortia

Two microbial consortia were used in this study. These consortia were originally collected from Kamajima soil, and enriched as a pentachlorophenol-dechlorinating anaerobic culture that contained *Archaea*, *Bacteroidetes* (*Parabacteroides*), *Firmicutes* (*Clostridia* including *Dehalobacter* as PCP-dechlorinating anaerobe), and *Spirochaetes* as major microorganisms [29,30]. Then, the dechlorinating culture was further subjected to enrichments using two different methods. One inoculum source (HL) was developed and maintained with humin by decreasing the concentration of Na-acetate from 10 mM to 0 mM and using NaHCO_3_ as a buffer and N_2_:CO_2_ (4:1) as the headspace gas, as reported previously [14]. The second inoculum source (HOA) was maintained with Kamajima soil at a constant concentration (10 mM) of Na-acetate, 3-*N*-morpholinopropanesulfonic acid (MOPS) as the buffer, and N_2_ as the headspace gas. The two microbial consortia, HL and HOA, were enriched from the HL and HOA inoculum sources, respectively, under the same conditions through two periods (I, II) (as illustrated in Figure S2). Briefly, these inocula were initially incubated in the basal medium with humin, NaHCO_3_ as buffer, and H_2_:CO_2_ (4:1) as the headspace gas in anaerobic vials as follows. The basal medium contained NH_4_Cl (1 g/L), MgCl_2_.6H_2_O (0.1 g/L), K_2_HPO_4_ (0.4 g/L), CaCl_2_.2H_2_O (0.05 g/L), NaHCO_3_ (4 g/L), Na-resazurin (0.1% *w/v*), trace element solution SL-10 (0.1% *v*/*v*) [31], and Se/W solution (0.1% *v*/*v*) [32]. Each of the above vials (with 120 mL) was added to 50 mL of the basal medium and 1 g of freeze-dried humin, and sparged with a mixed gas of N_2_ and CO_2_ (2:1, *v*/*v*) until the medium reached a pH of 7.0 ± 0.2. The vial was sealed with a black butyl rubber stopper and aluminum crimp caps, and autoclaved at 120 °C for 20 min. Prior to inoculation, filter-sterilized vitamin solution (0.5 mL) [33], pentachlorophenol (PCP) (20 µM, >90% purity), and Ti (III)-NTA 0.2 mM as reducing agent, were injected into the vial, and the headspace was further flushed with a mixture of H_2_ and CO_2_ (4:1, *v*/*v*) for 30 min. The inoculation was carried out at a ratio of 10% *v/v* of the previous culture. The culture was incubated at 30 °C for 18 d in the dark. After two generations, PCP was omitted from the medium to facilitate the enrichment of acetogens and methanogens. After the acetate production rate had stabilized, the enriched consortia, HL and HOA, were maintained and used for the study. The acetate and methane production during the development of the consortia are shown in Figures S3 (HL consortium) and S4 (HOA consortium).

### 2.3. Effect of Various Factors on Acetogenesis and Methanogenesis

#### 2.3.1. Experiment 1: Effect of Humin on Acetogenesis and Methanogenesis

A vial with a total volume of 60 mL was used in Experiments 1 and 2. The vial, containing 20 mL of basal medium and 0.4 g of humin, was sealed with black butyl rubber stopper and aluminum crimp caps, sparged with a mixture of N_2_ and CO_2_ (2:1, *v*/*v*) until the pH of the medium reached 7.0 ± 0.2, and then autoclaved at 120 °C for 20 min. After cooling, 0.2 mL of filter-sterilized vitamin solution and 0.2 mM Ti(III)-NTA were added to the vials, and the headspace was subsequently flushed with a mixture of H_2_/N_2_ and CO_2_ (4:1, *v*/*v*) for 20 min. The prepared vial was inoculated with 10% (*v*/*v*) of either consortia HL or HOA and then incubated at 30 °C in the dark (as illustrated in Figure S5). This procedure was also followed for Experiment 2.

The effect of humin on acetogenesis and methanogenesis was examined under five conditions (Table 1). In conditions 2, 3, 4, and 5, bicarbonate buffer was used with N_2_/CO_2_ sparging, and the headspace was flushed with H_2_/CO_2_ or N_2_/CO_2_ in the presence and absence of humin. In condition 1, the bicarbonate buffer was substituted with 20 mM MOPS and sparging and flushing of the headspace was performed with N_2_ alone.

#### 2.3.2. Experiment 2: Effect of Different Concentrations of FeS, NaCl, and MgSO_4_ on Acetogenesis and Methanogenesis

The effects of FeS, NaCl, and MgSO_4_ on acetate and methane production were examined under condition 2 and condition 4 (mentioned in Experiment 1) in which H_2_ was present and intact humin was either present or absent. The testing concentrations were 0.2, 0.5, and 2 g/L for FeS, 2, 6, and 12 g/L for NaCl, and 0.002, 0.02, and 0.2 g/L for MgSO_4_, respectively. FeS and NaCl were added to the medium as solid powders, and MgSO_4_ was added as 0.2 mL of an aqueous solution to obtain the desired concentration (as illustrated in Figure S6). To examine the chemical effect of FeS on CO_2_ fixation, the above-mentioned concentrations of FeS were also examined without the inoculum under conditions 2 and 4.

### 2.4. Chemical Analysis

H_2_ and methane were measured using a Shimadzu GC-14B gas chromatography system (Kyoto, Japan) equipped with a Molecular Sieve-5A column (60/80 mesh, 3 mm inner diameter and 2 m length) and a Porapak N column (50/80 mesh, 3 mm inner diameter, and 3 m length) in series, and a thermal conductivity detector (TCD) and a flame ionization detector (FID) in series, using N_2_ as a carrier gas at a flow rate of 20 mL/min. TCD was operated with helium as a reference gas at a flow rate of 20 mL/min, and FID was operated under a continuous supply of gases of H_2_ and compressed air at flow rates of 50 and 450 mL/min, respectively. The temperatures of the column, injector, and detector were 80 °C, 100 °C, and 100 °C, respectively.

For organic acid analysis, 1 mL of sample was taken from the culture, filtered through a 0.2 µm membrane filter (OmniporeTM, Merck, Darmstadt, Germany), and diluted with pure water at a ratio of 1:2 or 1:4 (*v*/*v*). The organic acids were determined by high-performance liquid chromatography (Shimadzu, LC-10AT, Kyoto, Japan) equipped with a Puresil C18 reversed-phase column (Waters, Milford, MA, US) and a UV detector at 210 nm. The column temperature was 40 °C. The mobile phase was 0.1% H_3_PO_4_ and the flow rate was 0.5 mL/min. The system was capable of detecting formate, acetate, propionate, butylate, and lactate. In this study, the detected organic acid was only acetate, which was determined by providing a calibration curve in the range from 0.1 mM to 10 mM using the standard of acetic acid (99.7%, Fujifilm Wako Pure Chemical Corporation, Osaka, Japan).

### 2.5. Microbial Community Analysis

After three transfers, 2 mL of HOA and HL consortia with humin were collected. Total genomic DNA was extracted using a FastDNA^TM^ Spin Kit for Soil (MP Biomedicals, Japan, Tokyo, Japan) according to the manufacturer’s protocol. The bacterial 16S rRNA genes were amplified by polymerase chain reaction (PCR) using the following primer pair: Pro341F (5′-CCT ACG GGN BGC ASC AG-3′) and Pro805R (5′-GAC TAC NVG GGT ATC TAA TCC-3′) [34]. The reaction mixtures contained 12.5 µL of KAPA HiFi HotStart Ready mix (KAPA Biosystems, Wilmington, MA, USA), 2.5 µL of each primer (2 µM), and 5 µL of template DNA (5 ng/µL). The PCR conditions were as follows: initial activation at 94 °C for 30 s, followed by 10 cycles at 94 °C for 10 s, 60 °C for 30 s, and 72 °C for 30 s, followed by 10 cycles at 94 °C for 10 s, 59 °C for 30 s, and 72 °C for 30 s, followed by 10 cycles at 94 °C for 10 s, 58 °C for 30 s, and 72 °C for 30 s, and a final extension at 72 °C for 4 min. The amplified 16S rRNA PCR products were purified using the AMPure XP kit (Beckman Coulter Genomics Inc., Brea, CA, USA) according to the manufacturer’s protocol, and were analyzed on a 1% agarose gel. The concentration of purified DNA was determined using a QuantiFluor dsDNA System (Promega Corporation, Fitchburg, WI, USA), and then sequenced using a MiSeq platform with a MiSeq reagent kit v3 (600 cycle, Illumina Inc., San Diego, CA, USA). A chimera check for the base sequences of each read was obtained from the analysis using USEARCH v6.1 [35]. Sequence reads with 99% similarity were classified into the same operational taxonomic unit (OTU) and OTU picking, and cluster analysis was performed using QIIME 2-2020-11 [36]. The OTUs were identified using the Silva database (version 138) [37].

### 2.6. Estimation of Electron Equivalents

Given that eight electrons are required to produce one mole of acetate or methane (Equations (1) and (2)), the electron equivalent donated from H_2_ or humin (µeeq/vial) was calculated by multiplying the amount of acetate produced from H_2_ or humin (µmole/vial) by 8. Given that each molecule of H_2_ contains two electrons, the electron equivalents from H_2_ consumed (µeeq/vial) were calculated by multiplying the amount of consumed H_2_ (µmole/vial) by 2.

### 2.7. Statistical Analysis

Statistical analysis was conducted by independent *t*-test for two variables and one-way analysis of variance (ANOVA) with Tukey’s post hoc test for more than two variables in IBM SPSS Statistics software package (version 21.0.0, IBM Corp., Armonk, NY, USA).

## 3. Results

### 3.1. Effect of Humin on CO_2_-Fixing Acetogenesis and Methanogenesis

**Figure 1:**
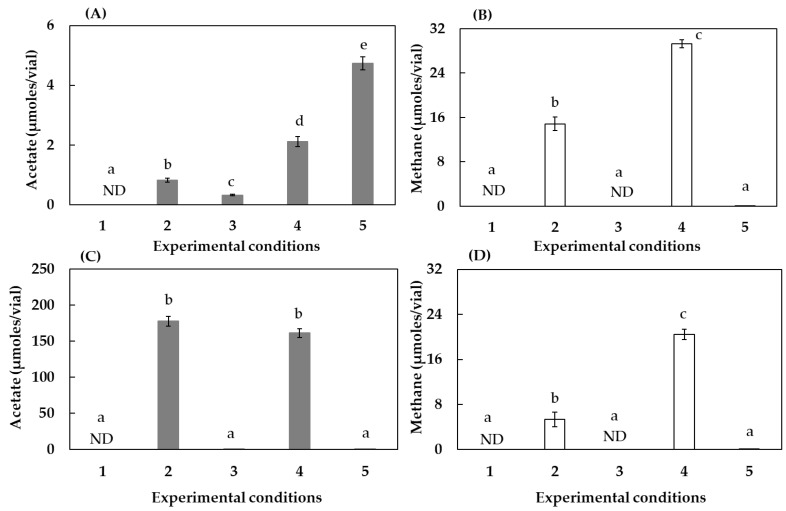
Effect of humin and H_2_ on the production of acetate and methane in the HL (**A**,**B**) and HOA (**C**,**D**) consortia, respectively. Conditions 1 to 5 are summarized in Table 1. Vertical bars represent the standard deviation. Different letters show the statistically significant difference (*p* < 0.05) by one-way ANOVA followed by Tukey’s post hoc test. ND: not detected.

As shown in Figure 1A and Figure 1B, in the presence of intact humin and the absence of other electron donors such as H_2_ (condition three), the HL consortium produced acetate but not methane, as reported in the previous paper [14]. The CO_2_ absence (condition one) resulted in no production of acetate, confirming that the HL consortium was a CO_2_-reducing acetogenic consortium. The utilization of extracellular electrons from humin for the reaction was confirmed by the higher production of acetate with reduced humin (condition five). The higher promotion in the reduced humin (condition five) than in the intact humin (condition three) suggested that the promotion level is dependent on the available electrons in humin. Although the HL consortium also utilized H_2_ as an electron donor for CO_2_-reducing acetogenesis (condition two), the reaction was further accelerated in the co-presence of humin (condition four), indicating that the HL consortium could use both H_2_ and extracellular electrons from humin for acetogenesis. Meanwhile, methane production was observed in the presence of H_2_ (condition two), but not in the presence of either intact or reduced humin (conditions three and five). However, methanogenesis was accelerated in the presence of H_2_ and humin (condition four), indicating that the utilization of extracellular electrons from humin for methanogenesis was limited by the co-presence of H_2_. We also examined whether acetate was utilized as an electron donor or a carbon source for methanogenesis in the HL consortium during incubation (Figure S7). The results showed that there was no change in the amounts of acetate and methane during the incubation, indicating that acetate degradation by acetoclastic methanogenesis did not occur in this consortium. Acetate was considered the end product.

The HOA consortium was also enriched as a CO_2_-fixing acetogenic and methanogenic consortium. The HOA consortium produced acetate at a much higher level than the HL consortium in the presence of H_2_ (Figure 1C, condition two). However, the HOA culture did not utilize extracellular electrons in humin (conditions three and five). The HOA consortium did not accelerate acetate production in the co-presence of H_2_ and intact humin (condition four) compared with the condition with H_2_ only (condition two). Meanwhile, the methanogenic activity was accelerated by the co-presence of H_2_ and humin (Figure 1D), which was similar to the activity in the HL consortium. Methanogenesis in the HOA consortium was considered to use extracellular electrons from humin only in the presence of H_2_.

The electron-donating capacities of H_2_ and humin were estimated in the HL consortium, as shown in Table 2. The electron-donating capacity of humin was estimated by assuming that the capacity of H_2_ did not change between conditions two and four. In acetogenesis, reduced humin had the highest electron-donating capacity (condition five). In the co-presence of H_2_ and humin (condition four), the estimated capacity of humin was higher than that of intact humin only (condition three). This suggested that intact humin was first biologically reduced and then utilized by the acetogenic bacteria, and the electrons from humin were more favorable than those from H_2_ for acetogenesis in the HL consortium. In methanogenesis, the estimated capacity of humin was approximately the same as that of H_2_. The preferable use of electrons from humin instead of H_2_ was also the case in methanogenesis, although humin alone cannot be the sole electron donor. Furthermore, regarding the electron balance, under condition two, the total electron equivalents supplied by H_2_ were higher than the total electron equivalents required for methane and acetate production, suggesting that there were unknown reactions which used H_2_ as an electron donor. Under condition four, the total electron equivalent supplied by H_2_ was lower than that estimated from methane and acetate production, indicating that humin was not only reduced by H_2_, but also induced to donate electrons to acetogens in the presence of H_2_.

### 3.2. Effect of Concentrations of FeS, NaCl, and MgSO_4_ on Acetogenesis and Methanogenesis

**Figure 2:**
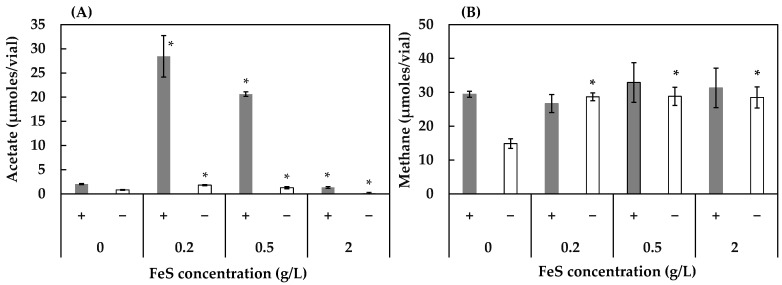
Effect of FeS concentration on the production of acetate (**A**) and methane (**B**) in the HL consortium in the presence (+) and absence (−) of humin. The conditions with 0 g/L of FeS are the same as conditions 4 (with humin) and 2 (without humin) in Figure 1. Vertical bars represent the standard deviation. The acetate and methane production with various FeS concentrations were statistically compared with those of 0 g/L of FeS using the independent *t*-test. * represents a significant difference at *p* < 0.05.

As shown in Figure 2A, the acetogenesis of the HL consortium was significantly accelerated with FeS, especially in the presence of humin, and the acceleration effect reached a maximum at 0.2 g/L of FeS. The process was slightly suppressed at 2 g/L FeS when compared to the condition without FeS addition. The addition of FeS increased approximately two-fold the methane production for the consortia without humin (irrespective of the FeS concentration), while for the consortia with the presence of humin, no significant change was observed (Figure 2B). No acetate or methane was detected in the absence of the inoculum (abiotic conditions) (data not shown), indicating that FeS did not chemically reduce CO_2_ to acetate or methane. These results suggest that FeS accelerated microbial acetogenic and methanogenic activities.

**Figure 3:**
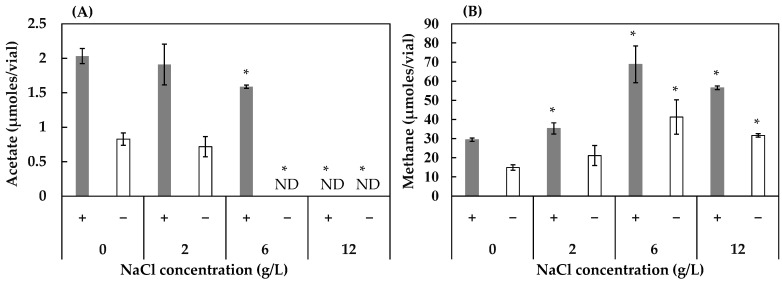
Effect of NaCl concentration on the production of acetate (**A**) and methane (**B**) in the HL consortium in the presence (+) and absence (−) of humin. The conditions with 0 g/L of NaCl are the same as conditions 4 (with humin) and 2 (without humin) in Figure 1. Vertical bars represent the standard deviation. Acetate and methane production with various NaCl concentrations were compared to those under the condition of 0 g/L of NaCl using the independent *t*-test. * represents a significant difference at *p* < 0.05. ND: not detected.

As shown in Figure 3A, the effect of NaCl on acetate production was detrimental for all concentrations, but more severe in the conditions without the humin. At 12 g/L of NaCl, both consortia with and without humin were completely inhibited. At the concentration of 6 g/L, humin addition enabled preservation of the majority (83%) of the acetate production when compared to the control without NaCl. In comparison to acetogenesis, NaCl addition increased methane production regardless of the presence/absence of humin, but with the maximum production at the concentration of 6 g/L (Figure 3B).

**Figure 4:**
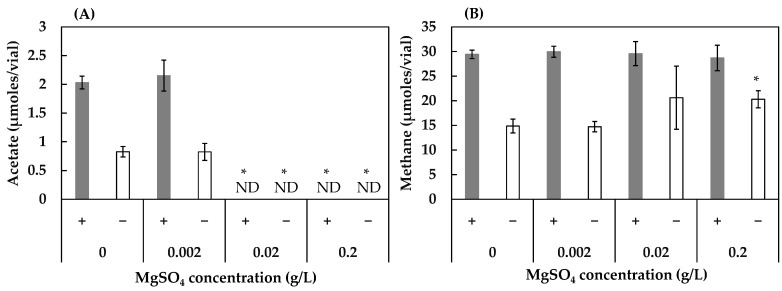
Effect of MgSO_4_ concentration on the production of acetate (**A**) and methane (**B**) in the HL consortium in the presence (+) and absence (−) of humin. The conditions with 0 g/L of MgSO_4_ are the same as the conditions 4 (with humin) and 2 (without humin) in Figure 1. Vertical bars represent the standard deviation. The acetate and methane production at various MgSO_4_ concentrations were compared to those under the condition of 0 g/L of MgSO_4_ using the independent *t*-test. * represents a significant difference at *p* < 0.05. ND: not detected.

The results obtained for the effect of MgSO_4_ revealed insignificant impact on both acetogenesis and methanogenesis, except concentrations 0.02 g/L and 0.2 g/L, which completely inhibited acetogenesis (Figure 4A), and 0.2 g/L, which slightly accelerated methane production in the absence of humin (Figure 4B).

**Figure 5:**
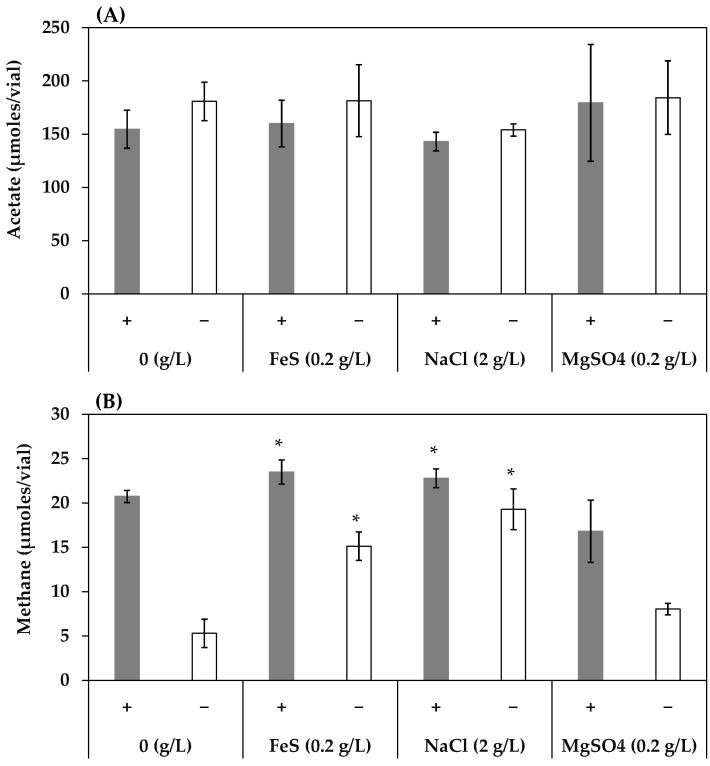
Effect of FeS (0.2 g/L), NaCl (2 g/L), MgSO_4_ (0.2 g/L) on the production of acetate (**A**) and methane (**B**) in the HOA consortium in the presence (+) and absence (−) of humin. The conditions with 0 g/L (indicating control) are the same as the conditions 4 (with humin) and 2 (without humin) in Figure 1. Vertical bars represent the standard deviation. The acetate and methane production at FeS (0.2 g/L), NaCl (2 g/L), MgSO_4_ (0.2 g/L) were compared to those under the condition of 0 g/L of FeS, NaCl, MgSO_4_ using the independent *t*-test. * represents a significant difference at *p* < 0.05.

These chemical effects were also examined in the HOA consortium. FeS (0.2 g/L), NaCl (2 g/L), and MgSO_4_ (0.2g/L) did not affect the acetogenesis of the HOA consortium, regardless of the presence of humin (Figure 5A). The difference in the effect of these chemical factors on the acetogenic activity of the HOA consortium compared with those in the HL consortium suggested that the acetogens differed between the two consortia, while the methanogenesis was promoted by FeS (0.2 g/L) and NaCl (2 g/L) (Figure 5B). The effects were more pronounced under the conditions without humin. MgSO_4_ (0.2 g/L) did not affect the methanogenesis, regardless of the presence of humin. That the trends in the effects of the chemical factors on the methanogenesis were almost the same suggested the same methanogens present in the two consortia.

### 3.3. Microbial Community Structure of HL and HOA Consortium

**Figure 6:**
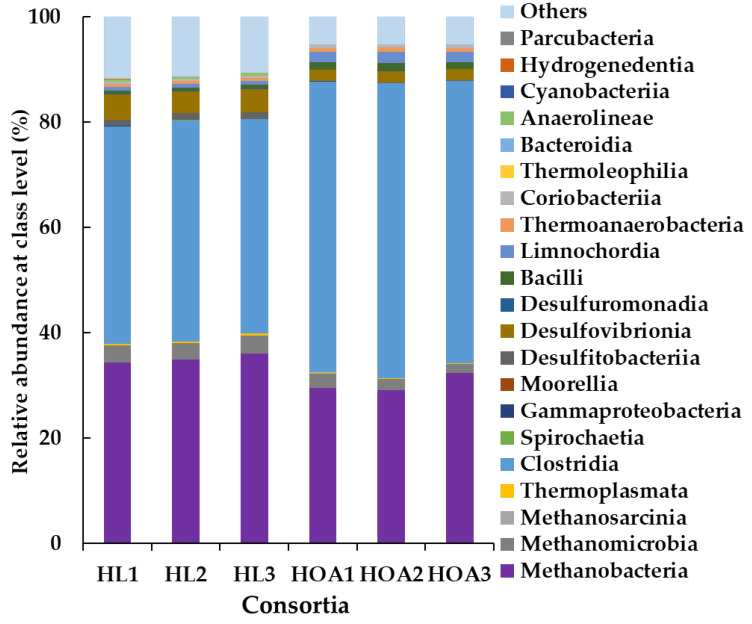
Microbial composition of the HL and HOA consortia, showing the major microorganisms (assessed in triplicate) based on 16S rRNA gene sequence.

The predominant classes in the HL consortium were *Clostridia* (41.4 ± 0.7%) and *Methanobacteria* (35.1 ± 0.9%), as shown in Figure 6. Most acetogens that reduce CO_2_ to acetate have been found in *Clostridia* [8,22,38]. *Methanobacteria* was composed of mostly the genus *Methanobacterium* (Figure S8), which is known as a hydrogenotrophic methanogen [39,40]. The major microbial composition of the HL consortium was almost the same as that of the HOA consortium, shown by the relative abundance at class level (Figure 6) and at genus level (Figure S8). Differences were observed in the minor microorganisms (Table 3) being detected in the HL consortium but not in the HOA consortium and vice versa. All the microorganisms listed in Table 3 belonged to the class *Clostridia*.

In addition, *Desulfovibrio* as sulfate-reducing bacteria [41], and *Geobacteraceae* as ferrous iron oxidizers [42], occupied 4.4 ± 0.3% and 0.1 ± 0.02% in the HL consortium, respectively, and were linked to the reactions with FeS and MgSO_4_.

## 4. Discussion

This study examined the effects of humin as an extracellular electron mediator and examined the effects of chemical and microbial factors on the activities of CO_2_-fixing acetogenesis and methanogenesis in consortia enriched from soil. The CO_2_-fixing acetate production in the HL consortium utilized either H_2_ or humin as an electron donor (Figure 1A). Both electron donors were utilized, but humin was a more preferable electron donor than H_2_ in the HL consortium (Table 2). Acetate formation utilizing H_2_ as an electron donor indicated acetogenesis using the Wood–Ljungdahl pathway for CO_2_ fixation. Intact humin extracted from paddy soil and not exposed to any reducing process still enabled CO_2_-fixing acetogenesis. These results are consistent with previous results [14]. The isoelectric potential of humin extracted from paddy soil has been estimated to be in the range of −300 mV to +272 mV vs. standard H_2_ electrode [16,43,44], and the standard redox potential at pH 7 (E_0′_) for acetate production from CO_2_ is −290 mV [22]. The intact humin used in this study would contain highly reduced fractions in the structure, more reduced than −290 mV (the standard redox potential of acetogenesis), and the highly reduced fraction drove the reaction. The versatility of the multiple electron donors observed in the HL consortium is also supported by previous reports on the characteristics of acetogens [11,45]. Meanwhile, the acetogens in the HOA consortium did not utilize the extracellular electrons of humin (Figure 1C), suggesting the presence of different acetogens.

Methane was produced both in the presence of H_2,_ or H_2_ and humin, in both HL and HOA consortia (Figure 1B,D). The same response to the different conditions in the two consortia suggested the same methanogens. Unlike acetogenesis, methanogenesis requires H_2_ for the utilization of extracellular electrons in the humin. The hydrogenotrophic methanogenesis pathway involves numerous complex enzymes as intermediates (e.g., ferredoxin (E_0′_ = −450 mV), F_420_ (E_0′_ = −340 mV), and CoM-S-SCoB (E_0′_ = −140 mV) [46,47]. A few of these enzymes in the pathway may not be able to utilize extracellular electrons from humin.

Sulfate appeared to be very inhibitory in acetate production but not in methane production in the HL consortium (Figure 4), where sulfate-reducing bacteria (e.g., *Desulfovibrio*) were detected in the HL consortium. Only the activity of acetogenesis in the HL consortium was completely inhibited in the presence of MgSO_4_ greater than 0.02 g/L. The acetogenesis of the HOA consortium was not affected by MgSO_4_ at 0.2 g/L (Figure 5). Methanogenic activity was not affected by all tested concentrations of MgSO_4_. In other studies [48,49,50,51], concentrations of MgSO_4_ over 0.02 g/L did not result in any effect on acetogenesis, demonstrating that the acetogenic communities in the HL consortium were highly sensitive to MgSO_4_.

In this study, FeS accelerated humin-utilizing CO_2_-fixing acetogenesis in the HL consortium (Figure 2). The acceleration of acetogenesis by FeS was optimal at 0.2 g/L and decreased at 0.5 g/L and 2 g/L. Abiotically, FeS did not produce any acetate. FeS can partially dissolve in the medium to provide Fe(II) and S(II) (sulfide), both of which can serve as electron donors for reduction reactions [52]. The interaction between anaerobic microbes and FeS has been observed previously [53]. *Geobacteraceae* has been shown to use Fe (II) as an electron donor for CO_2_ fixation [42], which was present in the HL consortium. Redox coupling between Fe (II) and CO_2_-reducing acetogenesis in microbial respiration would accelerate acetate production. Furthermore, Fe(II) and S(II) can also be electron sources either to reduce humin or to induce humin by donating electrons to CO_2_-fixing acetogens. This leads to increased acetate production in the presence of FeS and humin, as compared to that in the presence of FeS and absence of humin. However, the higher concentrations of FeS (0.5 g/L and 2 g/L) reduced the acetate production. This would be due to the higher concentration of sulfide in the medium, resulting in the negative effect on acetogenic activity. FeS also accelerated methanogenesis, especially in the conditions without humin, in both the HL and HOA consortia. FeS would be utilized as electron donor. The electron-donating effect of FeS has been reported in the methanogenic consortium under the condition buffered with bicarbonate [54].

NaCl produced an inhibitory effect on acetogenesis in the HL consortium and no effect in the HOA consortium, while accelerating methanogenesis in both consortia. Perhaps, acetogenic bacteria in these examined consortia did not utilize the Na^+^ motive force for acetogenesis [8] but rather the H^+^ motive force, while methanogenesis would use the Na^+^ motive force [25,26]. NaCl concentrations above 6 g/L inhibited acetogenesis. This inhibitory threshold was lower than that reported previously [55], in which *Clostridium carboxidivorans*, a CO_2_-fixing acetogen, was inhibited by NaCl at concentrations higher than 9 g/L. For methanogenesis, the optimal concentration of NaCl for the acceleration of methane production was 6 g/L.

The HL and HOA consortia were composed of almost the same microbial composition but the acetogenic activities differed in the efficiency, the utilization of extracellular electrons in humin, and the responses to the chemical factors. Since both consortia were obtained from Kamajima soil, the difference would be related to the preparation of the consortia. The microbial source of the HL consortium was maintained for 17 generations with basically 14 days of the incubation as one generation, under the conditions without an electron donor except for humin. Meanwhile, the microbial source for the HOA consortium was cultured with Na^+^-acetate for 16 generations, where one generation means the incubation for one month mostly (Figure S2). Then, the sources were subjected to the incubation under the conditions with H_2_ as inocula. The long-term maintenance of HL inoculum without either H_2_ or other electron donors would provide the characteristics of the HL consortium, while, the HOA consortium was maintained always with acetate or H_2_ during the preparation. This would result in H_2_-utilizing acetogenesis. That the structures of the major microbial composition in the HL and HOA consortia were almost the same also suggested that taxonomically dominated microorganisms would not be the responsible microorganisms functionally. There is increasing evidence that minor populations have an over-proportional role in biogeochemical cycles [56]. Higher activity has been observed in the rare species than in the abundant species [57]. It has been demonstrated that anaerobic phototrophic bacteria occupied about 0.3% of the total cell number but contributed more than 70% of the total uptake carbon in the system [58]. In this study, the minor population present only in the HL consortium were suggested as candidates for humin-utilizing acetogens. They were *Caloramator* (relative abundance: 1.86 ± 0.27%), *Hydrogenoanaerobacterium* (0.21 ± 0.03%), *Sedimentibacter* (0.03 ± 0.005%), etc. (Table 3), while *Caldicoprobacter* (0.08 ± 0.01%), *Sedimentibacter* (0.06 ± 0.01%), etc., were detected only in the HOA consortium, suggesting that these candidates were not utilizing humin but H_2_ as electron donor. Although these genera have not been reported to have CO_2_-fixing acetogenic activity, all of them belonged to the class *Clostridia*, in which most CO_2_-fixing acetogenic microorganisms have been reported [8,22,38]. It has also been pointed out that the rare microbes can influence the functionality of the abundant microbes [56]. The difference in the behavior of obtaining electrons between the HL and HOA consortia suggested that humin is not a universal electron donor for acetogens, but rather functions exclusively in a few microbial communities. Further study should be conducted to identify the humin-utilizing CO_2_-fixing acetogens in the HL consortium, with a particular focus on the minor population. In methanogenesis, humin was utilized as an extracellular electron donor only when H_2_ was present in both consortia, and NaCl demonstrated the ability to accelerate methanogenesis. *Methanobacterium* was detected commonly in both consortia and is also known to utilize the Na^+^ motive force [25], suggesting *Methanobacterium* as a major active methanogenic archaeon that produced methane from CO_2_.

The findings in this study would help understanding of the interaction of humin and examined chemical factors with CO_2_-fixing acetogenic and methanogenic activities. In subsurface environments, the multi-presence of humin and chemical factors (H_2_, FeS, MgSO_4_, NaCl) would have significant contribution to mitigating CO_2_ emissions from organic-matter oxidation.

## 5. Conclusions

The humin-assisted acetogens in the HL consortium were found to have the ability to co-utilize humin and H_2_ as electron donors for CO_2_-fixing acetogenesis, and appeared to preferably utilize the electrons from humin rather than from H_2_. Humin is not a universal electron donor for acetogens, as shown in the case of the HOA consortium. While the HOA consortium showed higher CO_2_-fixing acetogenesis utilizing H_2_ as electron donor, the ubiquitous distribution of humin and scarce presence of high H_2_ concentration suggest the importance of humin-assisted acetogenesis in the environment. In environmentally relevant chemical factors examined, FeS accelerated the acetogenesis of the HL consortium at 0.2 g/L as the optimal concentration, but MgSO_4_ inhibited at a concentration higher than 0.02 g/L. NaCl appeared to be inhibitory, suggesting that humin-assisted acetogens utilize the proton motive force. Comparative amplicon sequence analysis suggested minor groups in the HL consortium were the potential CO_2_-fixing acetogenic bacteria using extracellular electrons from humin. The CO_2_-fixing methanogenesis with *Methanobacterium* was accelerated by humin as an extracellular electron donor only when H_2_ was present. The methanogenesis was accelerated with NaCl at 6 g/L as the optimal concentration, with FeS in the range of 0.2–2 g/L without humin, while MgSO_4_ did not show any significant effect on methanogenesis.

## Figures and Tables

**Table 1:** Experimental set-up.

Condition	Intact Humin	Reduced Humin	Buffer	The Composition of Gas in the Headspace
1	+	−	MOPS	N_2_
2	−	−	HCO_3_^−^	H_2_/CO_2_
3	+	−	HCO_3_^−^	N_2_/CO_2_
4	+	−	HCO_3_^−^	H_2_/CO_2_
5	−	+	HCO_3_^−^	N_2_/CO_2_

**Table 2:** Electron-donating capacity of H_2_, of humin in acetate, and of methane production.

Condition	Electron Equivalents from H_2_ Consumed (µeeq/vial)	Electron Equivalents from H_2_ Used for Reducing Humin (µeeq/vial)	Electron Equivalents Required for Acetate Production (µeeq/vial)		Electron Equivalents Required for Methane Production (µeeq/vial)	
			From H_2_	From Humin	From H_2_	From Humin
2	176.8 ± 15.4	n/a	6.6 ± 0.5	n/a	118.9 ± 11.2	n/a
3	n/a	n/a	n/a	2.6 ± 0.2	n/a	0
4	241.2 ± 4.0	64.4 ± 18.2	6.6 ± 0.5	10.3 ± 0.9	118.9 ± 11.2	116.5 ± 5.9
5	n/a	n/a	n/a	37.9 ± 1.8	n/a	0

The values are shown as the mean ± standard deviation of triplicate measurements for each condition. Conditions 2–5 are summarized in Table 1. n/a: not applicable.

**Table 3:** Minor microbial groups detected only in one consortium of the HL and HOA consortia.

Genus ^a^	Relative Abundance (%)					
	HL1	HL2	HL3	HOA1	HOA2	HOA3
Hungateiclostridiaceae (uncultured bacterium) 1	0.01	0.01	0.01	0	0	0
Hungateiclostridiaceae (uncultured bacterium) 2	0.07	0.07	0.07	0	0	0
*Caloramator*	2.09	1.94	1.56	0	0	0
*Oxobacter*	0.01	0.01	0.01	0	0	0
Oscillospiraceae (no matching in genus level) 1	0.02	0.01	0.02	0	0	0
*Papillibacter*	0.01	0.01	0.01	0	0	0
*Hydrogenoanaerobacterium*	0.23	0.17	0.22	0	0	0
Oscillospirales (uncultured *Clostridium*) 2	0.05	0.05	0.04	0	0	0
*Anaerovorax*	0.03	0.01	0.02	0	0	0
*Sedimentibacter* 1	0.04	0.03	0.03	0	0	0
*Caldicoprobacter*	0	0	0	0.09	0.08	0.07
Oscillospirales (UCG-010) 3	0	0	0	0.01	0.01	0.01
*Sedimentibacter* 2	0	0	0	0.05	0.07	0.06

^a^ When genus is not identified, higher taxon (Family or Class, not in italic) is shown with the information on genus (uncultured bacterium, no matching with database in genus level, or uncultured group name). The same genus but different groups are numbered for disambiguation.

## Data Availability

The data presented in this study are available in supplementary materials, available online at https://www.mdpi.com/article/10.3390/ijerph19052546/s1.

## Supplementary Materials

The following are available online at https://www.mdpi.com/article/10.3390/ijerph19052546/s1: Table S1: Elemental composition of extracted humin; Figure S1: Fourier transform infrared (FTIR) spectra of extracted humin; Figure S2: Schematic diagram of development of microbial sources; Figure S3, Figure 4: Changes in the amount of acetate and methane during the development of HOA, HL microbial sources; Figure S5, Figure S6: Schematic diagram of Experiments 1, 2; Figure S7: Changes in the amount of acetate and methane during the incubation with acetate in the two consortia (HL and HOA); Figure S8: Microbial composition of the HL and HOA consortia at genus level.
