# Supplementary material for: Effect of Humin and Chemical Factors on CO2-Fixing Acetogenesis and Methanogenesis
Source: Int J Environ Res Public Health. 2022 Feb 22;19(5):2546. doi: 10.3390/ijerph19052546 (PMC8909181; doi:10.3390/ijerph19052546)
Supplement: Supplementary file 1 [file ijerph-19-02546-s001.zip › 220223-ijerph-1609744-Supplementary_Clean.pdf]

Supplemental Information for

Effect of Humin and Chemical Factors on CO_2-Fixing Acetogenesis and Methanogenesis

Biec Nhu Ha[1], Duyen Minh Pham[2], Takuya Kasai[1,2], Takanori Awata[3] and Arata Katayama[1,2*]

[1] Department of Civil Engineering, Graduate School of Engineering, Nagoya University, Chikusa, Nagoya 464-8603, Japan; ha.nhu.biec e5@s.mail.nagoya-u.ac.jp (B.N.H.); kasai.takuya@imass.nagoya-u.ac.jp (T.K.)

[2] Institute of Materials and Systems for Sustainability, Nagoya University, Chikusa, Nagoya 464-8603, Japan; pham.duyen.minh@imass.nagoya-u.ac.jp

[3] Graduate School of Engineering, Osaka Institute of Technology, Osaka, 535-8585, Japan; takanori.awata@oit.ac.jp

* Correspondence: katayama.arata@nagoya-u.jp; Tel.: +81-52-789-5856

Number of Pages: 7

Number of Figures: 8

Number of Tables: 1

Table S1. The elemental composition of extracted humin.

Elemental composition (%)					Ash (%)	C/N	H/C	O/C
C	H	N	O	S				
1.3	0.4	0.2	4.3	Under detection limit	93.9	10.6	3.2	2.4

Data represented as the mean of triplicates.

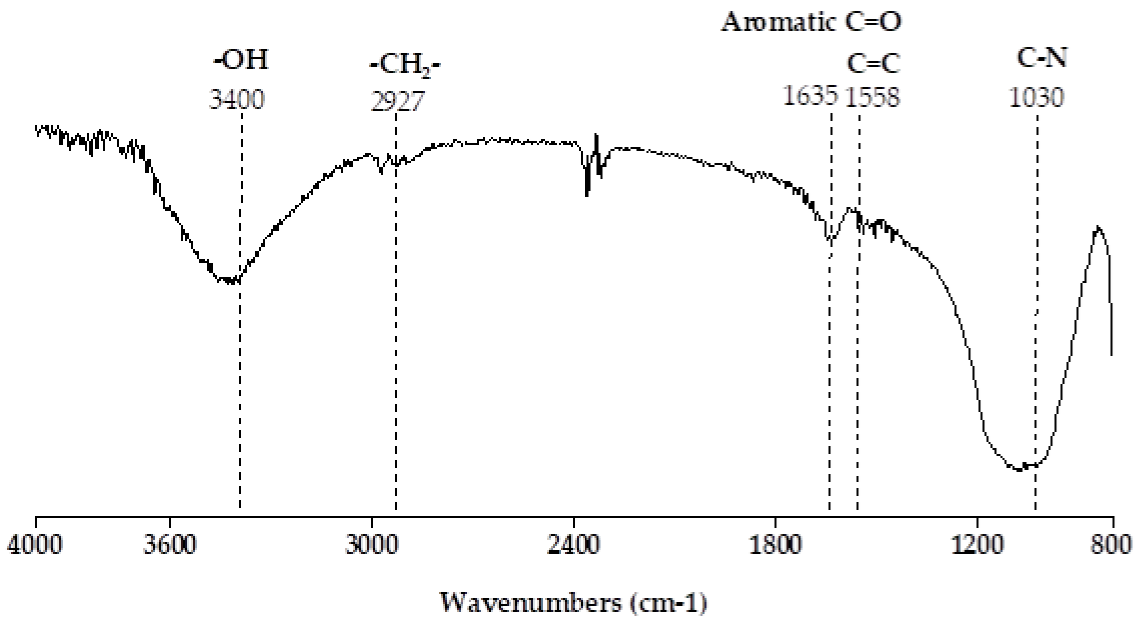

Figure S1. Fourier transform infrared (FTIR) spectra of extracted humin. Assignment of the peaks for the functional groups was referred from the studies of [1,2].

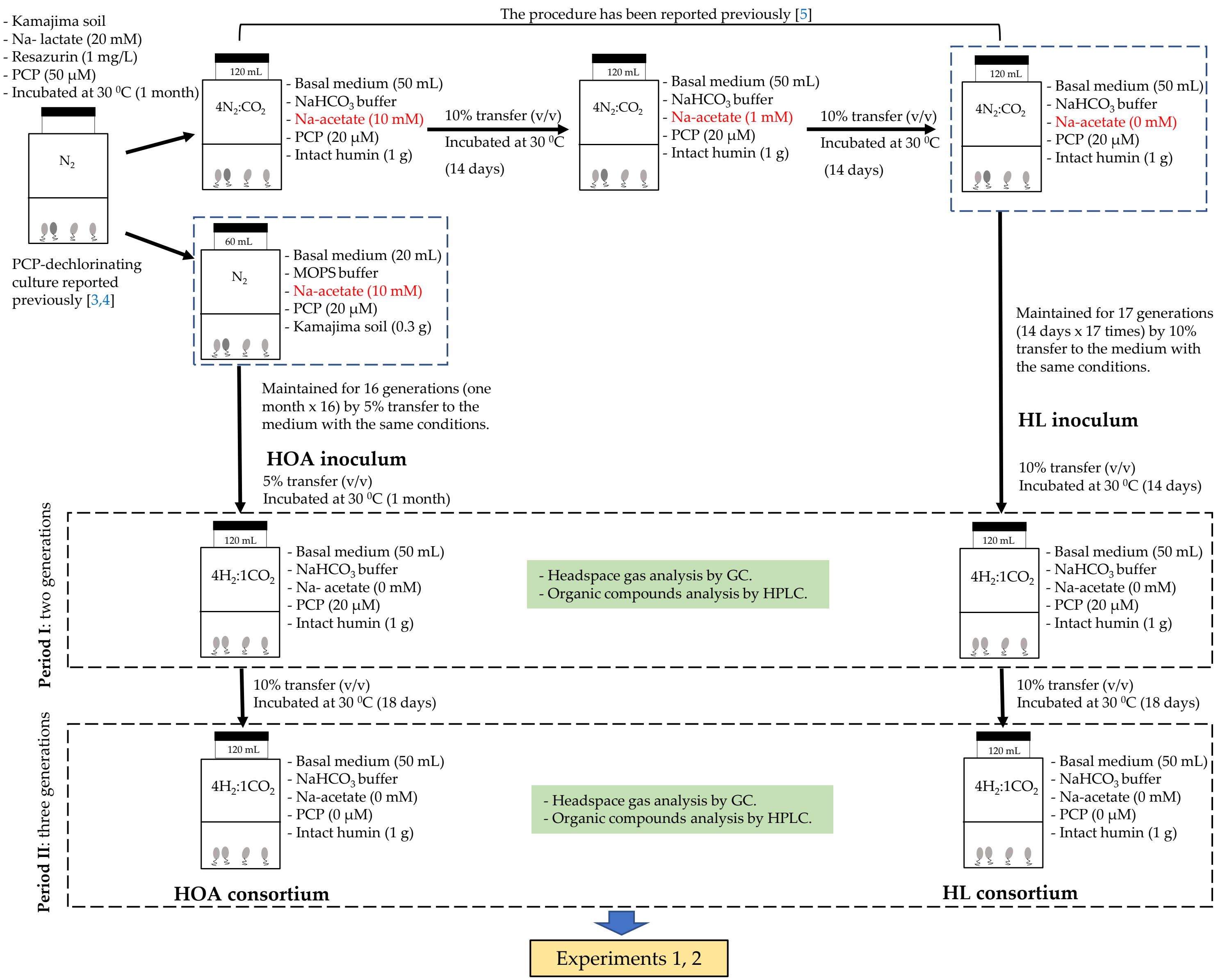

Figure S2. Schematic diagram of preparation of HL and HOA consortia. Soil and humin were autoclaved (121 °C, 15 min) prior to use.

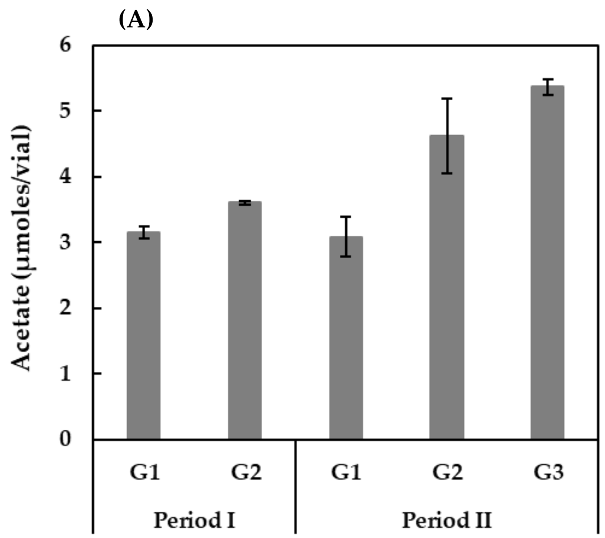

Figure S3. Changes in the produced amounts of acetate (A) and methane (B) during development of HL consortium. G1, G2, and G3 shows generation 1, 2, and 3, respectively. Periods I and II are shown in Figure S2.

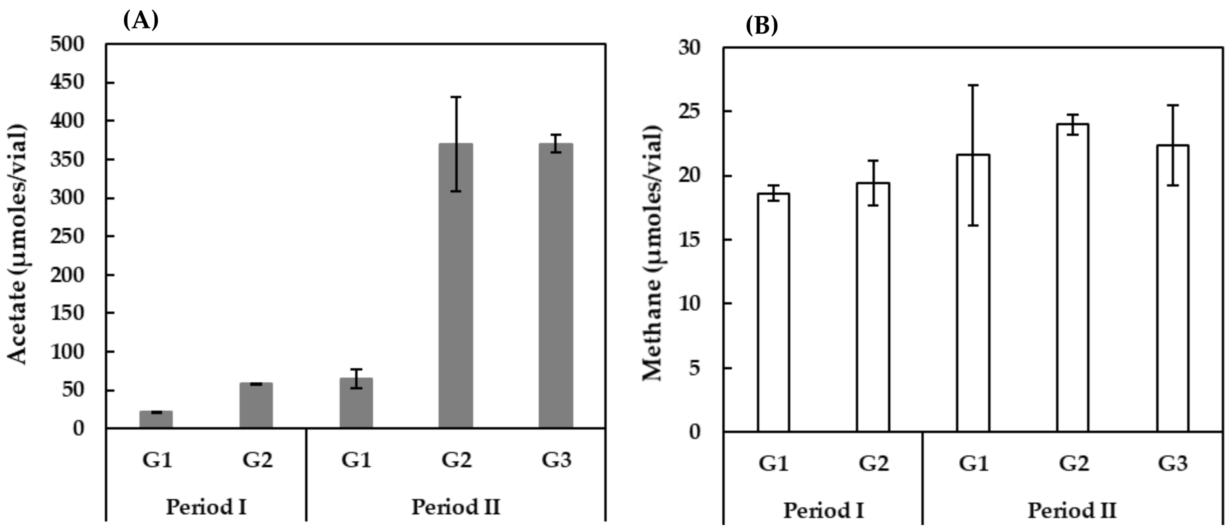

Figure S4. Changes in the produced amounts of acetate (A) and methane (B) during development of HOA consortium. G1, G2, and G3 shows generation 1, 2, and 3, respectively. Periods I and II are shown in Figure S2.

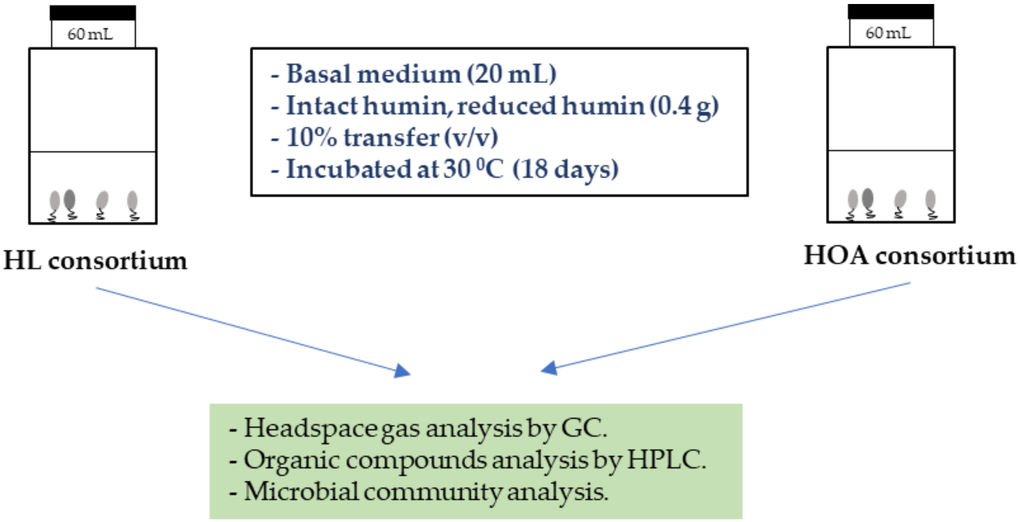

Figure S5. Schematic diagram of experiment 1.

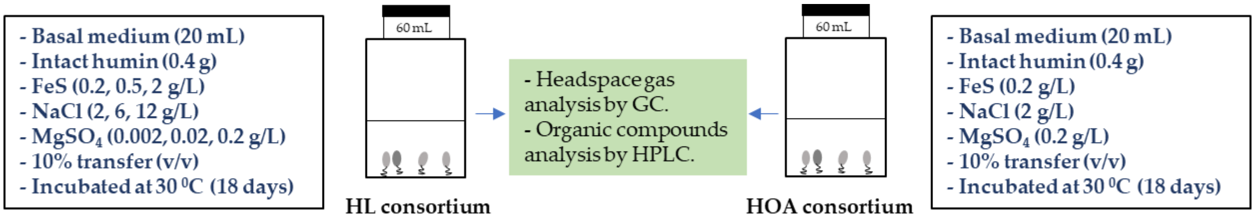

Figure S6. Schematic diagram of experiment 2.

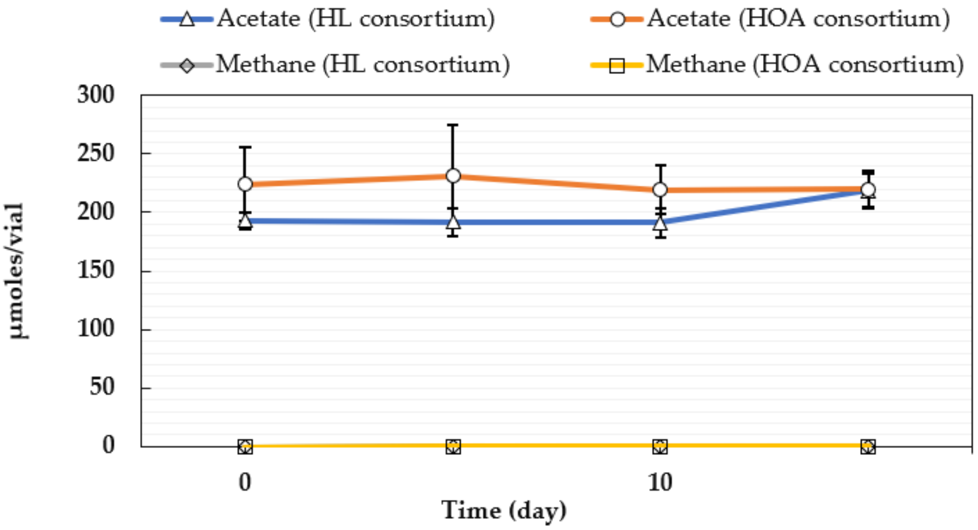

Figure S7. Changes in the amount of acetate and methane during incubation with acetate (220 µmoles/vial, 10 mM as initial concentration) and without another electron donor (hydrogen or humin) in two consortia (HL and HOA). No decrease in acetate and no increase in methane suggested no acetoclastic methanogenesis in the HOA culture. Vertical bars represent the standard deviation.

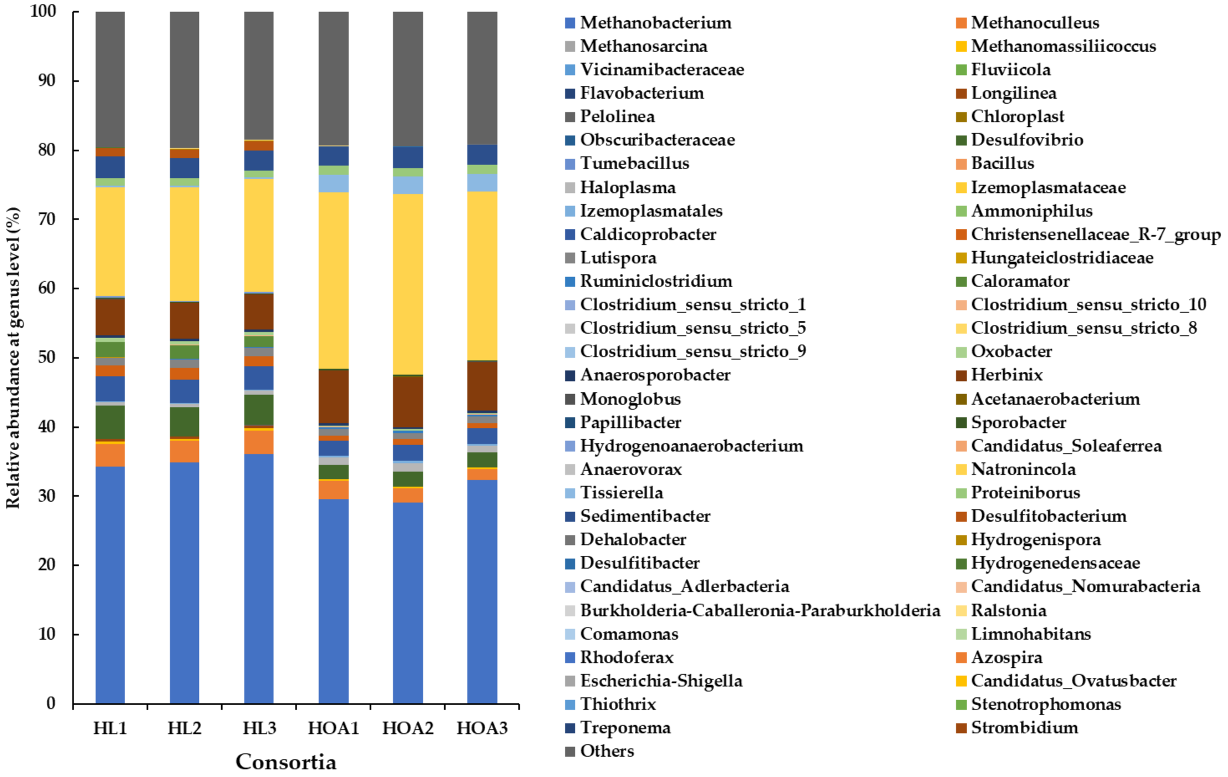

Figure S8. Microbial composition of the HL and HOA consortia at genus level. The others composed of uncultured, unidentified microbes.

References

1. Pham, M. D.; Katayama, A. Humin as an external electron mediator for microbial pentachlorophenol dechlorination: exploration of redox active structures influenced by isolation methods. Int. *J. Environ. Res. Public Health* **2018**, *15*, 2753.
2. Zhang, C.; Zhang, D.; Xiao, Z.; Li, Z.; Suzuki, D.; Katayama, A. Characterization of humins from different natural sources and the effect on microbial reductive dechlorination of pentachlorophenol. *Chemosphere* **2015b**, *131*, 110-116.
3. Yoshida, N.; Yoshida, Y.; Handa, Y.; Kim, H.K.; Ichihara, S.; Katayama, A. Polyphasic characterization of aPCP-to-phenol dechlorinating microbial community enriched from paddy soil. *Sci. Total Environ*. **2007**, *381*, 233–242.
4. Li, Z.; Inoue, Y; Suzuki, D.; Ye, L.; Katayama, A. Long-term anaerobic mineralization of pentachlorophenol in a continuous-flow system using only lactate as an external nutrient. *Environ. Sci. Technol*. **2013**, *47*, 1534-1541.
5. Laskar, M.; Kasai, T.; Awata, T.; Katayama, A. Humin assists reductive acetogenesis in absence of other external electron donor. *Int. J. Environ. Res. Public Health* **2020**, *17*, 4211.
